# Global Evidence on the Association between Cigarette Graphic Warning Labels and Cigarette Smoking Prevalence and Consumption

**DOI:** 10.3390/ijerph15030421

**Published:** 2018-02-28

**Authors:** Anh Ngo, Kai-Wen Cheng, Ce Shang, Jidong Huang, Frank J. Chaloupka

**Affiliations:** 1 Department of Economics, the University of Illinois at Chicago, Chicago, IL 60607, USA; ango4@uic.edu; 2 Institute for Health Research and Policy, the University of Illinois at Chicago, Chicago, IL 60608, USA; cshang@uic.edu (C.S.); fjc@uic.edu (F.J.C.); 3 School of Public Health, Georgia State University, Atlanta, GA 30303, USA; jhuang17@gsu.edu; 4 Division of Health Policy and Administration, School of Public Health, the University of Illinois at Chicago, Chicago, IL 60608, USA

**Keywords:** graphic warning labels (GWLs), cigarette smoking, cigarette consumption

## Abstract

*Background*: In 2011, the courts ruled in favor of tobacco companies in preventing the implementation of graphic warning labels (GWLs) in the US, stating that FDA had not established the effectiveness of GWLs in reducing smoking. *Methods*: Data came from various sources: the WHO MPOWER package (GWLs, MPOWER policy measures, cigarette prices), Euromonitor International (smoking prevalence, cigarette consumption), and the World Bank database (countries’ demographic characteristics). The datasets were aggregated and linked using country and year identifiers. Fractional logit regressions and OLS regressions were applied to examine the associations between GWLs and smoking prevalence and cigarette consumption, controlling for MPOWER policy scores, cigarette prices, GDP per capita, unemployment, population aged 15–64 (%), aged 65 and over (%), year indicators, and country fixed effects. *Results*: GWLs were associated with a 0.9–3 percentage point decrease in adult smoking prevalence and were significantly associated with a reduction of 230–287 sticks in per capita cigarette consumption, compared to countries without GWLs. However, the association between GWLs and cigarette consumption became statistically insignificant once country indicators were included in the models. *Conclusions*: The implementation of GWLs may be associated with reduced cigarette smoking.

## 1. Introduction

Graphic warning labels (GWLs) have been shown to be an effective tobacco control policy in providing health information to the public [1,2,3,4,5] and in increasing the knowledge of health risks of smoking [5,6]. In the past decade, many countries have implemented GWLs. Article 11 of the WHO Framework Convention on Tobacco Control (FCTC), with 180 parties to the treaty, calls for countries to adopt pictorial labels on cigarette packages, with warnings covering at least 50% coverage in the principal display area [7]. 

By 2016, there were 105 countries or jurisdictions requiring pictorial warnings, and 77 countries or jurisdictions had implemented such warnings [8]. In 2016, 94 countries or jurisdictions also adopted the requirement of at least 50% coverage of the front and back of cigarette packages [8]. Even though GWLs have been adopted in many countries, the implementation of GWLs in the US is still under debate. In 2011, the US Food and Drug Administration (FDA) issued regulations requiring tobacco companies to add GWLs to cigarette packs. However, the regulations were challenged by tobacco companies. The Courts ruled in favor of tobacco companies in preventing the implementation of the regulations, in part stating that FDA had not established the effectiveness of GWLs in reducing smoking.

In recent years, growing evidence demonstrates that GWLs are more effective in decreasing smoking than text-only warnings [4,9,10,11,12]. GWLs have been found to attract greater attention and greater recall of health warning messages among rural male smokeless tobacco users than text-based warnings [12]. Moreover, larger GWLs have been documented to affect many smoking-related outcomes [13,14,15,16,17,18]. For example, a recent study conducted an experiment to examine the impact of GWLs on visual attention among low-SES smokers and at-risk youth in the US and found that larger GWLs (50% versus 30%) increased visual attention to the warnings and to the pictorial content [18]. A number of other studies on the impacts of GWLs on smoking-related outcomes in Canada—where GWLs with at least 50% coverage have been implemented since 2001 also documented that GWLs reduced smoking prevalence and increased quit attempts [19,20] and that larger GWLs were more effective in delivering the health risks of smoking [4]. 

Despite growing evidence on the impacts of GWLs on smoking-related outcomes, few studies investigate the effectiveness of GWLs on actual smoking behaviors, especially across countries. Huang et al. employed a quasi-experimental methodology to investigate the effect of GWLs in Canada in 2000 and found that GWLs significantly reduced smoking rates in Canada compared to the US and that the implementation of GWLs would have reduced smoking rates in the US by 12–20% [21]. 

This paper contributes to the literature by adding evidence on the impacts of GWLs on actual smoking behaviors across countries. This study takes advantage of global datasets from over 60 countries to examine the associations between GWLs and countries’ smoking-related outcomes, controlling for economic conditions and tobacco control policy environments across countries. This study provides global evidence on the associations between GWLs and adult smoking prevalence and cigarette consumption. To the best of our knowledge, this paper is the first to examine the association between GWLs and cigarette consumption. 

## 2. Materials and Methods 

### 2.1. Data Sources 

#### 2.1.1. The WHO MPOWER Package

The information on country-level GWLs, tobacco control policies, and cigarette prices was taken from the WHO MPOWER package. Information on cigarette GWLs was obtained from the question “Do the health warnings on packages include a photograph or graphic?” in the WHO MPOWER 2007–2008, 2010, 2012, and 2014 datasets [5,22,23,24]. A dichotomous variable for cigarette GWLs was constructed, with countries that had GWLs coded as 1 and those that did not coded as 0. To fill out the missing values of the dichotomous variable in years of 2009, 2011, and 2013, we identified the effective dates of cigarette GWLs for each country by reviewing Euromonitor International cigarette and tobacco country reports, ERC reports, the Tobacco Labeling Resource Centre website, and other online resources [25,26,27]. Based on these effective dates, we coded the dichotomous variable of GWLs as 0 for the years before the effective dates and as 1 for the years after these dates.

The information on cigarette prices during the 2007–2014 period was also obtained from the WHO MPOWER package. Cigarette prices were defined as price of a 20-cigarette pack of the most sold brand international dollars (at purchasing power parity) in years of 2007–2008, 2010, 2012, and 2014. To fill out the missing values of prices for years 2009, 2011, and 2013, we interpolated cigarette prices using data from previous years. 

The WHO MPOWER package further contained the information on the six proven tobacco control measures: M (monitor tobacco use), P (protect people from smoke), O (offer help to quit), W (warn about the dangers of tobacco), E (enforce bans on tobacco marketing), and R (raise taxes on tobacco) [28]. For each policy dimension, a score of 1 represented a lack of data and a score of 2–4 for M measure and a score of 2–5 for POWER represented the weakest to the strongest policy strength in the years 2007–2008, 2010, 2012, and 2014 [28]. The six MPOWER scores measure both the lack of data and the strengths of each policy dimension. A categorical variable to indicate whether a country has missing data (i.e., score of 1) was created for each MPOWER measure. Since the values of six policy scores were missing for years of 2009, 2011, and 2013, we filled in the missing values of these scores by using the same scores of previous years assuming that there were no policy changes across these years. 

#### 2.1.2. Euromonitor International Tobacco and Cigarette Country Reports 

Data on smoking prevalence and cigarette consumption was obtained from Euromonitor International Tobacco and Cigarette Country Reports [25]. The reports included the information on smoking prevalence for 63 countries in the 2007–2014 period. The reports defined adult smokers as daily smokers who were older than the minimum legal sales smoking age in the country [25]. Smoking prevalence was thus the percentage of daily smokers in the population. 

Following Ng et al. [29], we constructed cigarette consumption in a country as a sum of retail and illicit cigarette sales. According to the reports’ definitions, legal retail sales were duty-paid, machine manufactured white-stick products and did not include duty-free sales [25]. Illicit trade cigarettes were defined as non-duty paid cigarettes including smuggled and counterfeit/fake products combined [25]. Per capita cigarette consumption was then defined as the ratio of the total cigarette consumption and the number of the population aged 15 and over. 

#### 2.1.3. World Bank Database 

The information on countries’ demographic characteristics such as GDP per capita, unemployment, % population aged 15–64, and % population aged 65 and over was obtained from the World Bank database [30]. Country-level GDP per capita was calculated in international dollars and converted to real terms using consumer price index. Population aged 15–64 and population aged 65 and over were the percentage of the population that was in these age ranges [30]. Unemployment rates were defined as the share of the labor force that was available for work and was seeking employment but was without work [30]. 

The datasets were aggregated and linked using year and country identifiers. The final analytical samples were restricted to countries with full information on smoking prevalence, cigarette consumption, and other independent variables. The final analytical samples included 490 country-year observations from 63 countries in the smoking prevalence sample and 593 country-year observations from 75 countries in the cigarette consumption sample. Approximately 49% and 51% of the countries in the smoking prevalence analyses were high-income countries (HICs) and low and middle income countries (LMICs) respectively. Similarly, approximately 47% and 53% of the countries in the cigarette consumption analyses were high-income countries and low and middle income countries. The income categorization was based on what income category a country was in for most years in the samples. High income and low and middle income countries were defined based on the definitions of the World Bank database. HICs were countries with ≥$12,236 GDP per capita and LMICs were countries with <$12,236 GDP per capita [30].

### 2.2. Methods 

Fractional logit regressions and OLS regressions were employed to examine the associations between cigarette GWLs and cigarette smoking prevalence and consumption respectively. The reason that we employed fractional logit regressions was because we measured smoking prevalence at the country level using the percentages between 0 and 1. In our regression models, we controlled for cigarette prices, six MPOWER scores, country-level GDP per capita, population aged 15–64 (%), and population aged 65 and over (%). Since the six MPOWER scores measure both the lack of data and the strengths of each policy dimension, indicators of missing values of each score within each MPOWER measure were included in the models to separate out the effect of missing data from the effect of the policies. To tease out any time-invariant and country-invariant specific factors that may affect cigarette use, we further controlled for year and country fixed effects in the models respectively. Two-way fixed effects models—a method that expands the difference-in-difference approach to repeated treatments in multiple time periods were also employed to examine the association between GWLs and smoking prevalence and cigarette consumption [31,32]. By controlling for both year and country fixed effects in the model, we only used with-in country changes over time in the implementation of GWLs for model identification. 

To examine any potential multi-collinearity that may yield unexpected signs or implausible magnitudes of the estimates in the two-way fixed effects models [33], we further estimated Variance Inflation Factor (VIF) by investigating to what extent GWLs were highly correlated with other independent variables. Our VIF estimates were 6.62 for the smoking prevalence analysis and 5.96 for the cigarette consumption analysis. These numbers are below 10-the rule-of-thumb [34], indicating that the multi-collinearity is a lesser concern here. The standard errors were clustered at the country level to adjust for any inter-temporal correlations. All analyses were performed in Stata Version 13.0 (College Station, TX, USA).

## 3. Results

**Figure 1:**
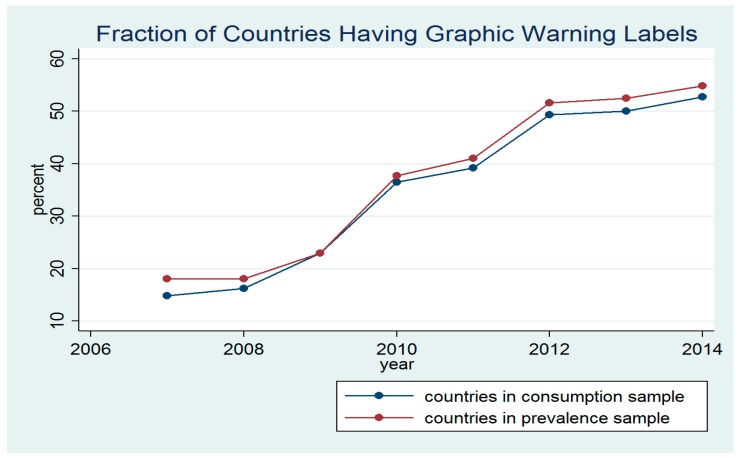
The fraction of countries having graphic warning labels (GWLs) during the study period (2007–2014) in the smoking prevalence and cigarette consumption samples.

Figure 1 presents the fraction of countries that had GWLs in the two analytical samples. The final analytical samples included countries with full information on smoking prevalence, cigarette consumption, and other independent variables. As Figure 1 indicates, in 2007 approximately 15% and 18% of the countries in the cigarette consumption and smoking prevalence analytical samples had GWLs in cigarette packages. The fraction of countries having GWLs increased significantly during 2009–2012 period, reaching approximately 55% in 2014.

Table 1 contains summary statistics for both analytical samples calculated using all the years in the study period. The mean of smoking prevalence among adults was 24.9%. The average per capita cigarette consumption per year was 1487 sticks, equivalent to approximately 75 packs of cigarette per year or 6 packs of cigarette per month. 37% and 35% of the countries in two analytical samples had GWLs at some point during 2007–2014. The average score of each policy dimension in six MPOWER measures was between 3 and 4. This implies that while some countries implemented these tobacco control policies with a medium strength, some implemented at a higher strength and that there was still much room for improvement. The average price of a 20-cigarette pack of the most sold brand at international dollars was 4.184 in international dollars (purchasing power parity) in the smoking prevalence analyses and 3.934 in international dollars (purchasing power parity) in the cigarette consumption analyses. 

Table 2 presents the estimates of the associations between GWLs and smoking prevalence and cigarette consumption, estimated using fractional logit regressions and OLS regressions. Model 1 controls for cigarette price, GDP per capita, and countries’ demographic characteristics. Model 2 further controls for year indicators. Model 3 controls for both year indicators and countries’ tobacco policy environment (MPOWER scores) and Model 4 further controls for country fixed effects. 

While the upper panel shows the coefficients of interest, the lower panel shows marginal effects, percentage changes due to GWLs, and elasticities of interest (price and income elasticities). The estimates of the association between and GWLs and smoking prevalence are statistically insignificant in all models and marginal effect ranges from −0.009 to −0.03. 

In cigarette consumption model, results indicate that GWLs were significantly associated with reduced cigarette consumption. Countries with GWLs experienced a reduction of 230 to 287 sticks of per capita cigarette consumption. However, when country indicators were included in the regression, the association became not statistically significant. 

Price elasticities of smoking prevalence range from −0.074 to −0.134 and statistically insignificant. On the other hand, price elasticities of cigarette consumption range from −0.349 to −0.410 and statistically significant. Similarly, countries’ GDP per capita was significantly associated with increased cigarette consumption. The estimate of income elasticity for cigarette consumption in the model was 0.363 (*p* < 0.01), indicating that a 10% increase in income was associated with a 3.63% increase in cigarette demand. 

## 4. Discussion

In this study, using the data from over 60 countries during the 2007–2014 period, we examined the associations between GWLs and smoking prevalence and cigarette consumption and provided global evidence on the impacts of GWLs on these smoking outcomes. About 36–37% of countries in the analytical samples had GWLs at some point during the study period. To the best of our knowledge, our study is the first to examine the association between GWLs and cigarette consumption. Our results suggest that GWLs were associated with a 0.9–3 percentage point decrease in adult smoking prevalence. However, this association is not statistically significant. GWLs were significantly associated with reduced cigarette consumption. Countries with GWLs experienced a reduction of 230–287 sticks of per capita cigarette consumption compared to countries without GWLs. However, the association becomes statistically insignificant when country indicators are included in the model. 

We found that the price elasticity for overall cigarette demand ranges from −0.467 to −0.484. This is consistent with the findings of other studies which found that price elasticities for overall cigarette demand tend to fall within a relatively wide range from −0.14 to −1.23, with most falling in a narrower range of −0.3 to −0.5 [35]. Our price elasticity estimate of −0.484 is also close to the finding of another study which found an average of price elasticity of cigarette demand of −0.48 [36]. 

With a price elasticity of cigarette consumption in a range of –0.349 to –0.410, this implies that a 10% increase in cigarette prices is associated with a decrease of 3.49% to 4.10% in cigarette consumption. In terms of income elasticity, we did not find any significant association between income and smoking prevalence. However, we found a significant association between income and cigarette consumption. The results of our models suggest an income elasticity of 0.363 (*p* < 0.01). This result implies that a 10% increase in income would increase cigarette consumption by 3.63%. 

Two alternative specifications were performed to examine the robustness of the results. A linear probability model was employed to re-examine the association between GWLs and smoking prevalence and a linear interpolation of MPOWER scores was employed to fill out the missing values of MPOWER scores. Results were very similar to those of our models, indicating our findings are robust to different model specifications. 

Our study is an ecological study with outcome variables (i.e., smoking prevalence and cigarette consumption) measured at the country level. This study has some limitations. First, while GWLs have been shown to have differential effects on different subpopulations [11,18,37], our study could not confirm this argument by investigating the impact of GWLs on different subpopulations using the aggregate data at the national level. Second, smoking prevalence was measured as a single outcome variable. Thus, we could not separate the impact of GWLs on smoking behaviors in terms of initiation and cessation. Further studies may consider to employ individual-level data to investigate the effect of GWLs on these smoking outcomes to provide detailed effectiveness of GWLs. Third, with a limited sample size of 35 high income countries and 40 low and middle income countries in both analytical samples, we did not have enough statistical power to examine the differential effect of GWLs on cigarette smoking across different income levels. 

Despite these limitations, our study has included as many countries as possible. In addition, we have controlled for other tobacco control policies and economic conditions, as well as year indicators and country fixed effects in the models to capture time-invariant and country-invariant specific factors that may affect cigarette smoking. 

## 5. Conclusions

Using the data from over 60 countries during the 2007–2014 period, we investigated the associations between GWLs and smoking prevalence and cigarette consumption and provided global evidence on the impacts of GWLs on these smoking outcomes. Our paper is also the first one to examine the association between GWLs and cigarette consumption. Our results suggest that the implementation of GWLs may be associated with reduced cigarette smoking. Thus, countries without GWLs may consider implementing GWLs. 

## Figures and Tables

**Table 1:** Summary statistics (2007–2014).

Variables	Analytical Samples			
	Smoking Prevalence		Per Capita Cigarette Consumption	
	Mean	SD	Mean	SD
Smoking Prevalence	0.249	0.079		
Per capita cigarette consumption (in thousand sticks)			1.487	0.888
Graphic warning labels (GWLs)	0.369	0.483	0.351	0.478
M score	3.345	0.825	3.204	0.936
Missing M score	0.020	0.142	0.059	0.236
P score	2.814	1.245	2.786	1.204
Missing P score	0.098	0.298	0.094	0.293
O score	3.837	0.690	3.789	0.761
Missing O score	0.00	0.00	0.012	0.108
W score	3.360	0.960	3.251	1.016
Missing W score	0.00	0.00	0.012	0.108
E score	3.500	0.932	3.251	1.016
Missing E score	0.00	0.00	0.012	0.108
R score	4.065	0.786	3.499	0.985
Missing R score	0.00	0.00	0.012	0.108
Rescaled GDP per capita	2.372	1.552	3.911	0.934
MPOWER price	4.184	2.211	3.934	2.159
Unemployment rate	8.189	5.144	8.739	5.777
Population aged 15–64 (%)	67.007	3.319	66.665	4.661
Population aged 65 and over (%)	12.580	5.426	11.293	5.802
Number of country-year obs.	490		593	
Number of Countries	63		75	

*Note*: sample sizes and country composition were different for smoking prevalence and cigarette consumption analytical samples due to data availability. The summary statistics were calculated using all the years in the study period. M: Monitor tobacco use; P: Protect people from smoke; O: Offer help to quit; W: Warn about the dangers of tobacco; E: Enforce bans on tobacco marketing; R: Raise taxes on tobacco; GDP: Gross domestic product.

**Table 2:** The association between graphic warning labels and adult smoking prevalence and cigarette consumption across alternative specifications.

Dependent Variables	Smoking Prevalence				Cigarette Consumption			
	Model 1	Model 2	Model 3	Model 4	Model 1	Model 2	Model 3	Model 4
Graphic warning labels (GWLs)	−0.067	−0.046	−0.160	−0.005	−0.26 **	−0.23 *	−0.287 *	−0.09
	(0.071)	(0.073)	(0.118)	(0.025)	(0.090)	(0.096)	(0.127)	(0.058)
GDP per capita	−0.066	−0.069	−0.054	−0.03	−0.057	−0.058	−0.078	0.138 **
	(0.049)	(0.050)	(0.052)	(0.056)	(0.057)	(0.058)	(0.058)	(0.05)
MPOWER Cigarette Price	−0.043	−0.039	−0.042	−0.024	−0.143 **	−0.139 **	−0.132 **	−0.155 ***
	(0.031)	(0.032)	(0.035)	(0.016)	(0.041)	(0.042)	(0.042)	(0.028)
Year Indicators	N	Y	Y	Y	N	Y	Y	Y
Country Fixed Effects	N	N	N	Y	N	N	N	Y
MPOWER scores	N	N	Y	Y	N	N	Y	Y
Number of observations	490	490	490	490	593	593	593	593
Number of countries	63	63	63	63	75	75	75	75
Marginal Effect	−0.012	−0.009	−0.030	−0.001	−0.260 **	−0.23 *	−0.287 *	−0.09
Percent Changes due to GWLs	−4.82%	−3.6%	−12.05%	−0.40%	−17.48% *	−15.47% *	−19.3% *	−6.05%
Marginal effect for price	−0.008	−0.007	−0.008	−0.004	−0.143 **	−0.139 **	−0.132 **	−0.155 ***
Price elasticity	−0.134	−0.118	−0.13	−0.074	−0.378 **	−0.368 **	−0.349 **	−0.410 ***
Income elasticity	−0.116	−0.124	−0.095	−0.052	−0.150	−0.153	−0.205	0.363 **

*Note:* Standard errors in parentheses. * *p* < 0.01, ** *p* < 0.05, *** *p* < 0.001. Standard errors were clustered at the country level. Other covariates controlled in the regressions are unemployment rate, percent population aged 15–64, percent population aged 65 and over, and indicators of missing MPOWER scores (if applicable). The marginal effect of GWLs was calculated using the command ‘margins, dydx()’ in Stata. The marginal effect was then used to calculate the % change in smoking prevalence at means based on the formula: (marginal effect/the average of smoking prevalence or cigarette consumption).
